# Supplementary figures and images for: Phage infection and sub-lethal antibiotic exposure mediate Enterococcus faecalis type VII secretion system dependent inhibition of bystander bacteria
Source: PLoS Genet. 2021 Jan 7;17(1):e1009204. doi: 10.1371/journal.pgen.1009204 (PMC7790226; doi:10.1371/journal.pgen.1009204)

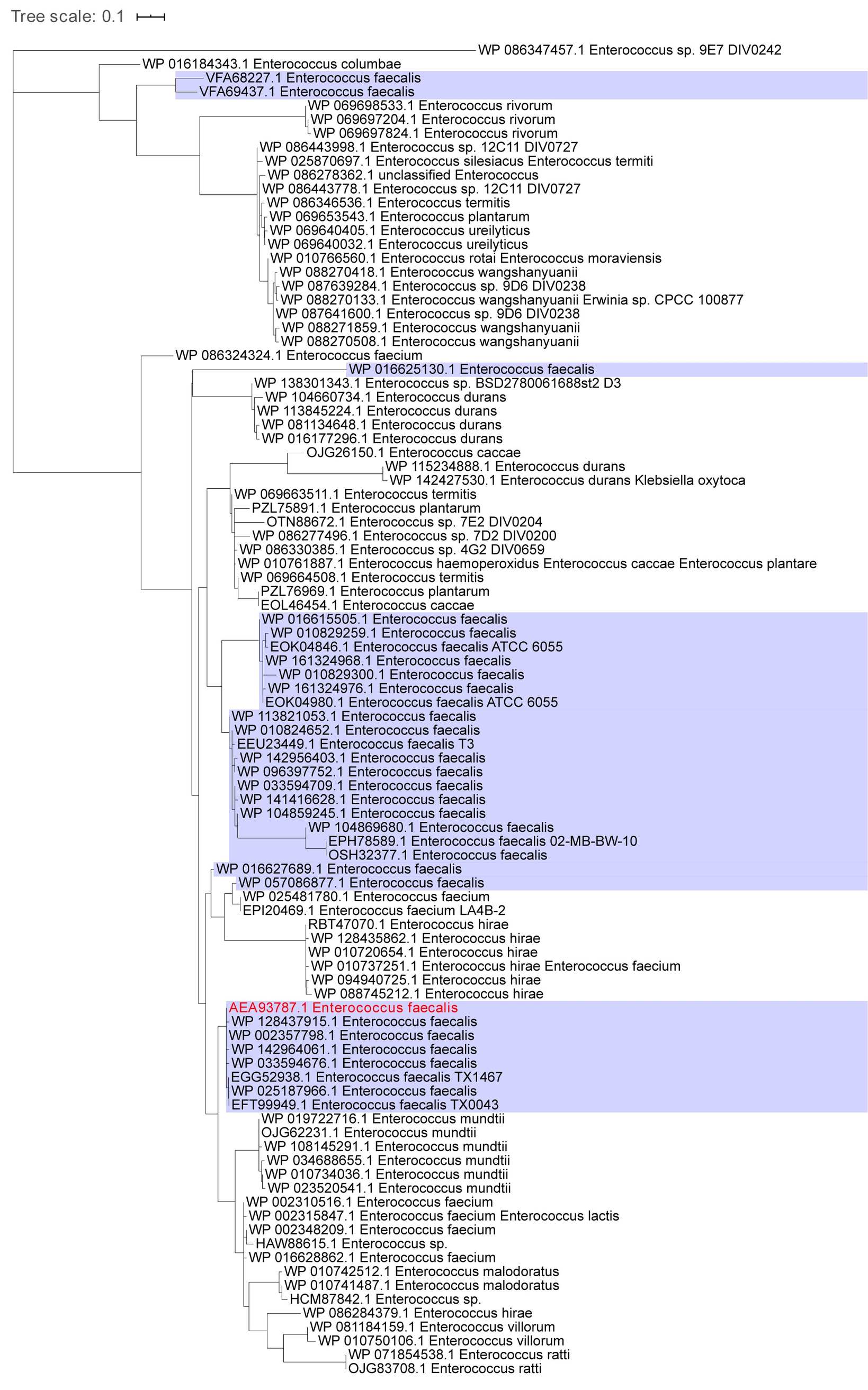

Supplement: S1 Fig — Non-redundant sequences (n = 96) were identified using NCBI BLAST with OG1RF EsxA (OG1RF_11100) as the input. The tree was constructed in MEGAX using the Maximum Likelihood method and JTT matrix-based model and is drawn to scale, with branch lengths measured in the number of substitutions per site. The tree with the highest log likelihood (-3544.39) is shown. E. faecalis sequences are highlighted in purple, and the GenBank identifier for EsxA from OG1RF (AEA93787.1) is shown in red font. (TIF) [file pgen.1009204.s001.tif]

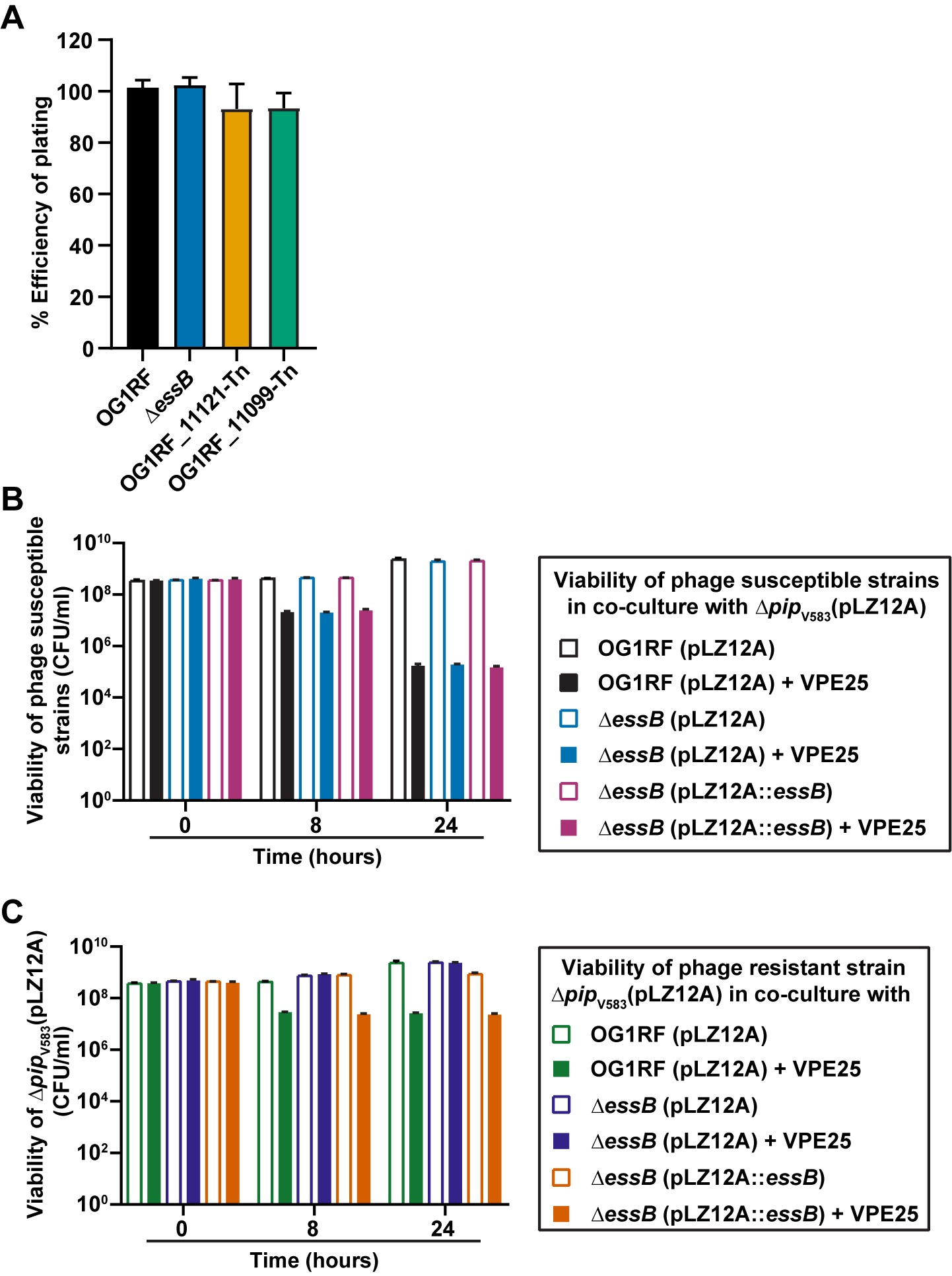

Supplement: S2 Fig — (A) The measurement of phage particles released from wild type E. faecalis OG1RF, ΔessB, OG1RF_11121-Tn, and OG1RF_11099-Tn mutant strains following phage VPE25 infection. (B) Viability of strains of the OG1RF background exhibiting differential T7SS activity in the absence and presence of phage. (C) Viability of T7SS susceptible strains during intraspecies competition experiments in the absence and presence of phage. Data represent three biological replicates. Error bars indicate standard deviation. *P < 0.0001 by unpaired Student’s t-test. (TIF) [file pgen.1009204.s002.tif]

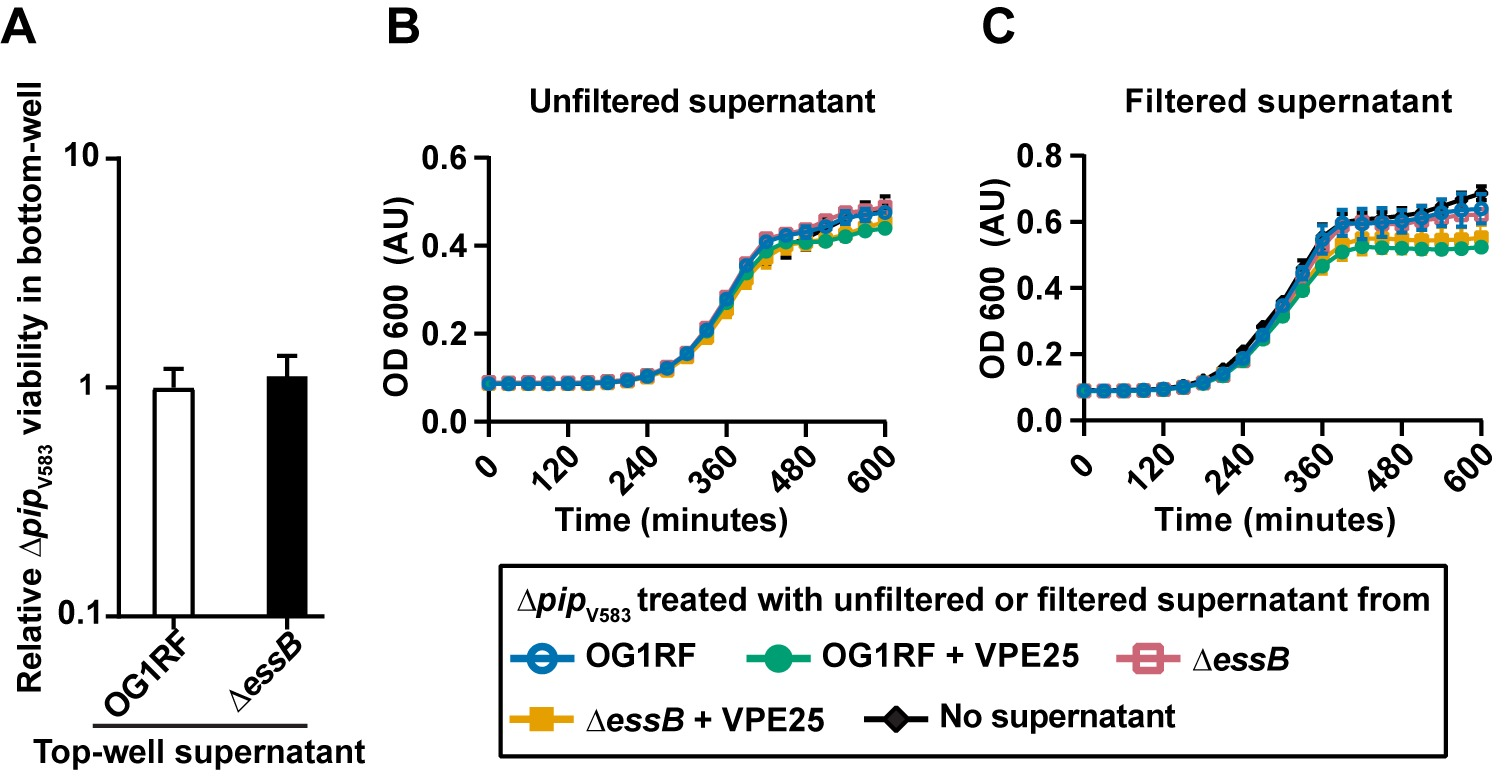

Supplement: S3 Fig — Intraspecies competition experiment performed in the presence of unfiltered supernatant from phage treated and untreated E. faecalis wild type OG1RF or ΔessB added (A) to the top of a well separated by a 0.4 μm membrane from the bottom well containing E. faecalis ΔpipV583 culture, and bacterial viability was determined after 24 hours, or (B) directly into E. faecalis ΔpipV583 culture in microtiter plate wells (P = 0.7955 by two-way analysis of variance [ANOVA]). (C) Growth of ΔpipV583 was monitored in the presence of filtered supernatant from uninfected and phage infected cultures of wild type E. faecalis OG1RF and ΔessB (P = 0.0883 by two-way analysis of variance [ANOVA]). E. faecalis ΔpipV583 cultures in all of these three contact-dependent assays contained gentamicin (25 μg/ml) to prevent growth of the OG1RF background strains that may have carried over in unfiltered supernatants. Error bars indicate standard deviation. (TIF) [file pgen.1009204.s003.tif]

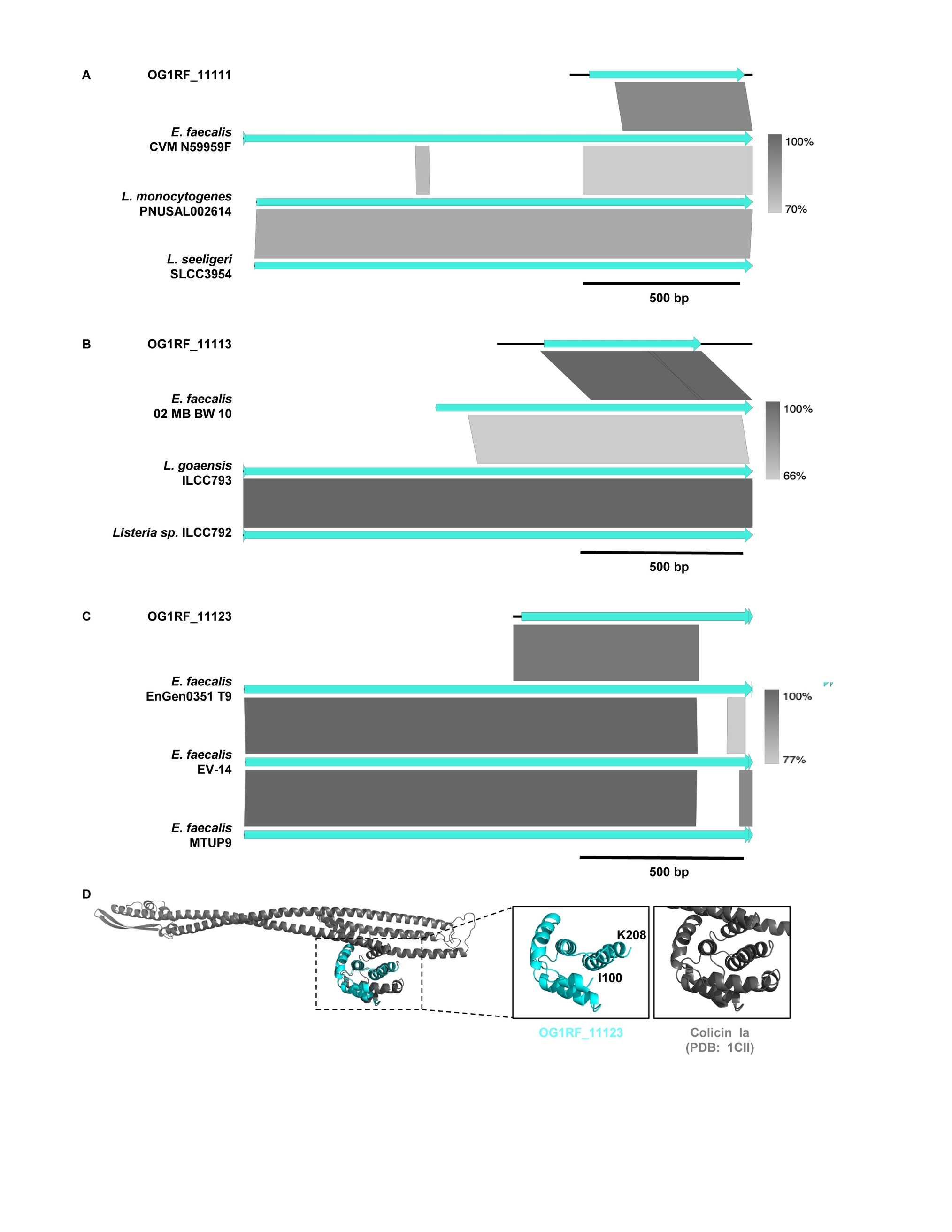

Supplement: S4 Fig — OG1RF_11111, OG1RF_11113, and OG1RF_11123 sequences were used as input for NCBI BLAST. Alignments and homology were rendered in EasyFig. (TIF) [file pgen.1009204.s004.tif]

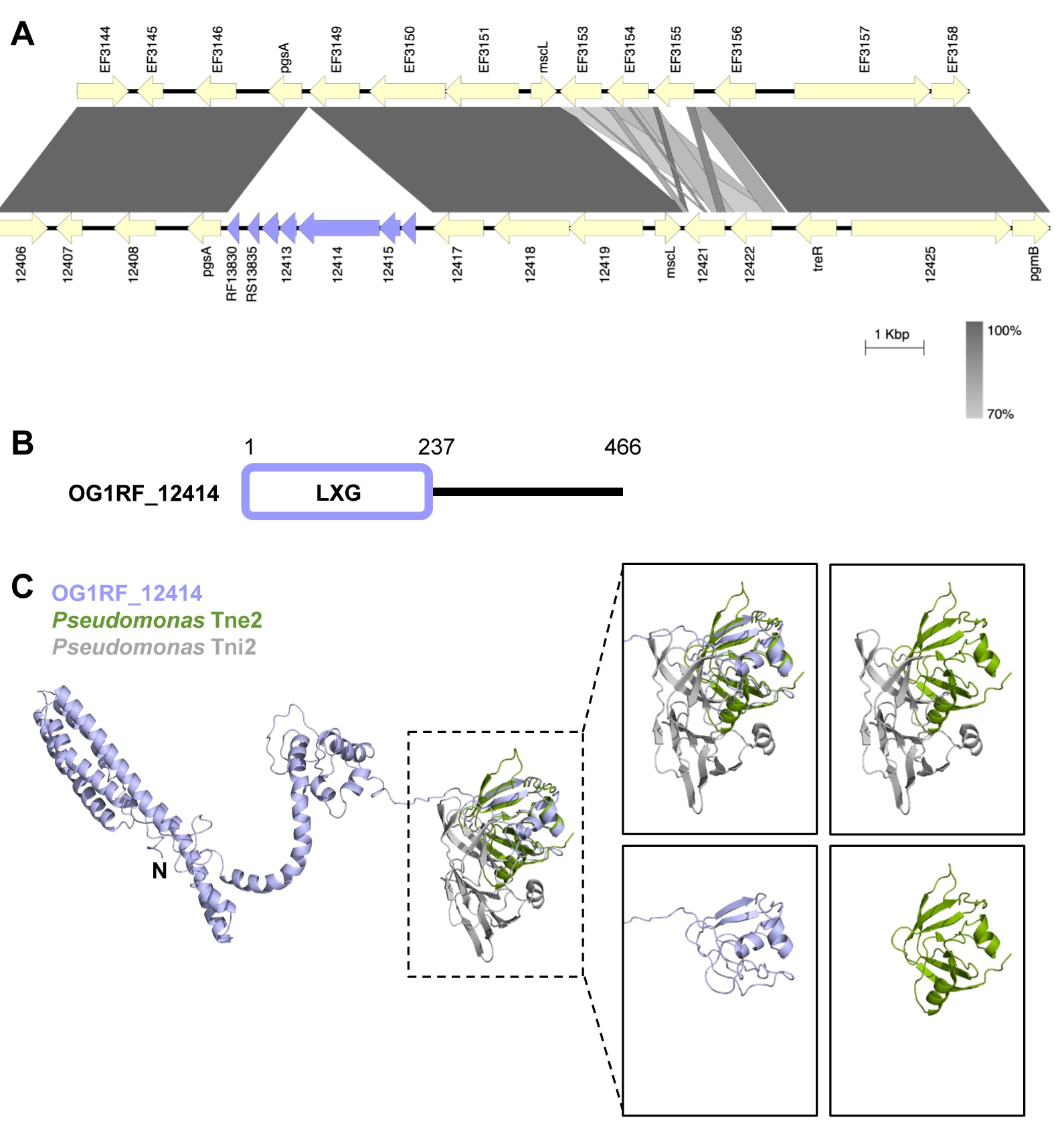

Supplement: S5 Fig — (A) Schematic showing homology between V583 (NC_004668.1, top) and OG1RF (NC_017316.1, bottom). Sequences were obtained from NCBI, and homology comparisons were rendered in EasyFig. (B) Cartoon depicting the LXG domain of OG1RF_12414 (identified using KEGG and ExPASy PROSITE). (C) Predicted structural homology between OG1RF_12414 (lilac) and the Pseudomonas protogens Pf-5 Tne2/Tni2 complex (PDB 6B12). Tne2 is shown in green, and Tni2 is shown in gray. Structural modeling was done using PHYRE2, and images were rendered in Pymol. (TIF) [file pgen.1009204.s005.tif]

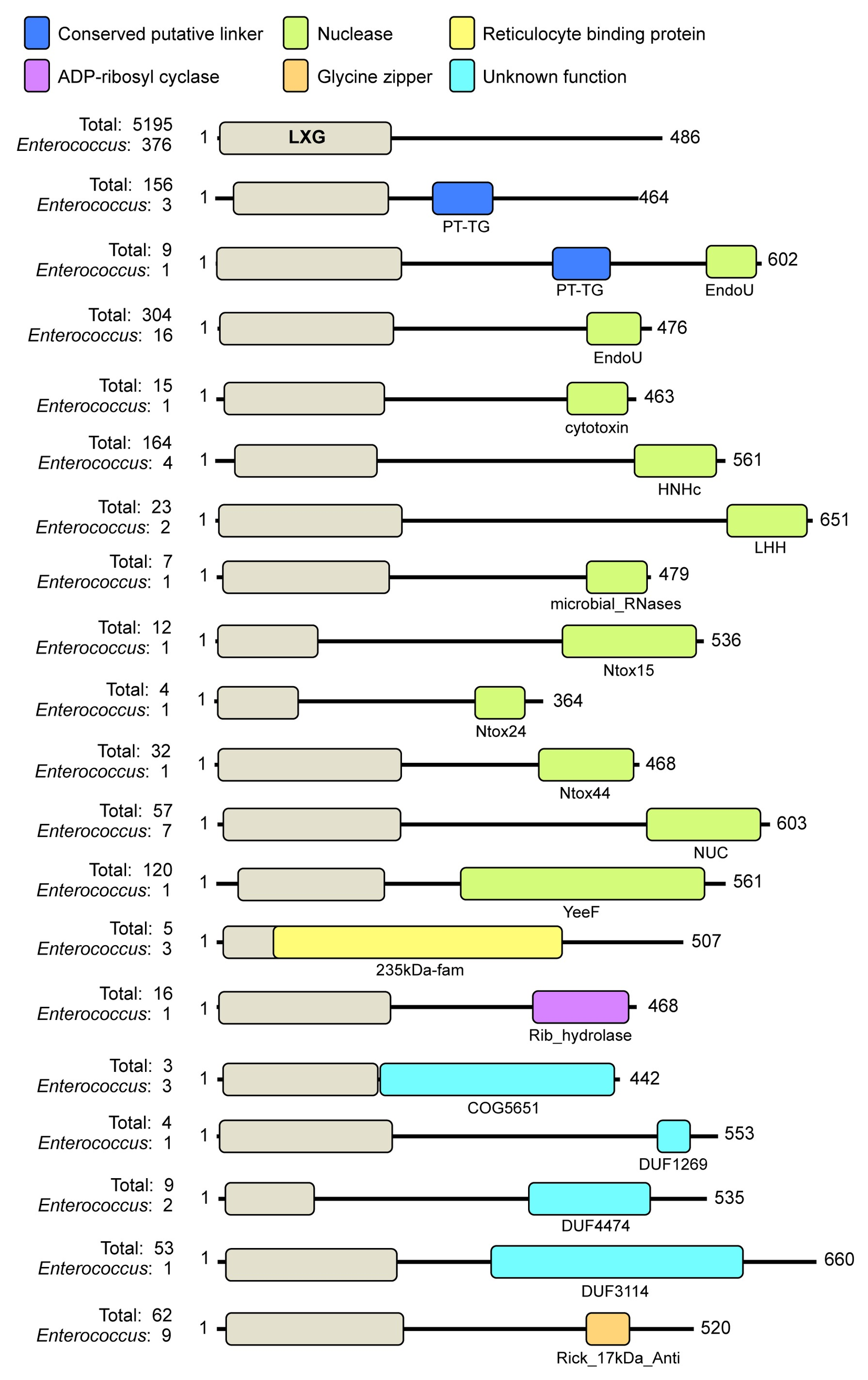

Supplement: S6 Fig — Domain architectures were identified using the NCBI Conserved Domain Architectural Retrieval Tool (DART) with OG1RF_11109 as an input. Diagrams are drawn to scale. (TIF) [file pgen.1009204.s006.tif]

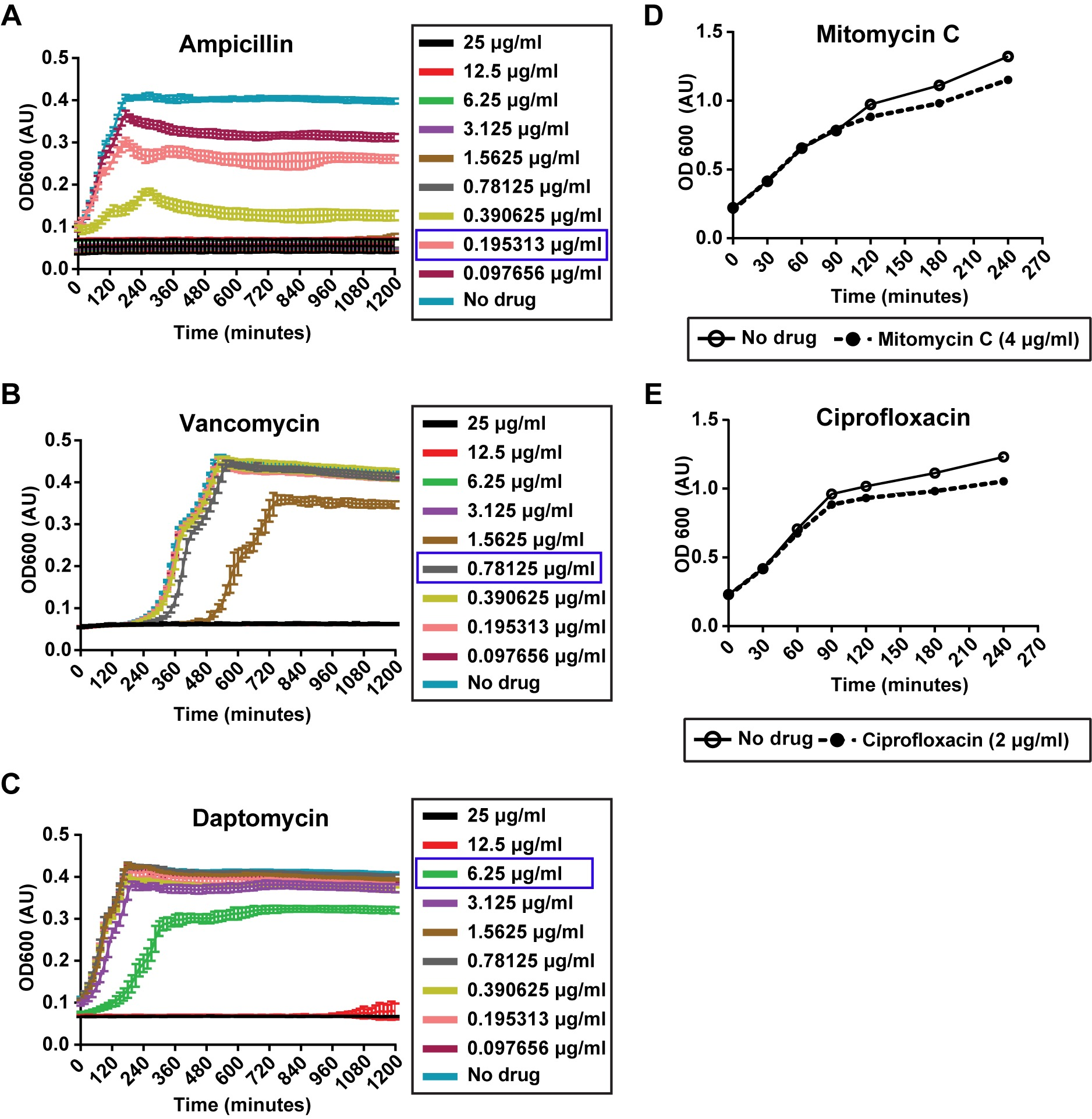

Supplement: S7 Fig — Growth of wild type E. faecalis OG1RF was monitored over 20 hours in the presence or absence of (A) ampicillin, (B) vancomycin and (C) daptomycin in microtiter plates. The antibiotic concentrations highlighted with a blue box were deemed sub-inhibitory and used to investigate T7SS gene expression levels. Early log-phase cultures of E. faecalis OG1RF were grown in the presence or absence of (D) mitomycin C (4 μg/ml) or (E) ciprofloxacin (2 μg/ml) to show that these concentrations of DNA targeting antibiotics do not prevent bacterial growth. Error bars indicate standard deviation. (TIF) [file pgen.1009204.s007.tif]

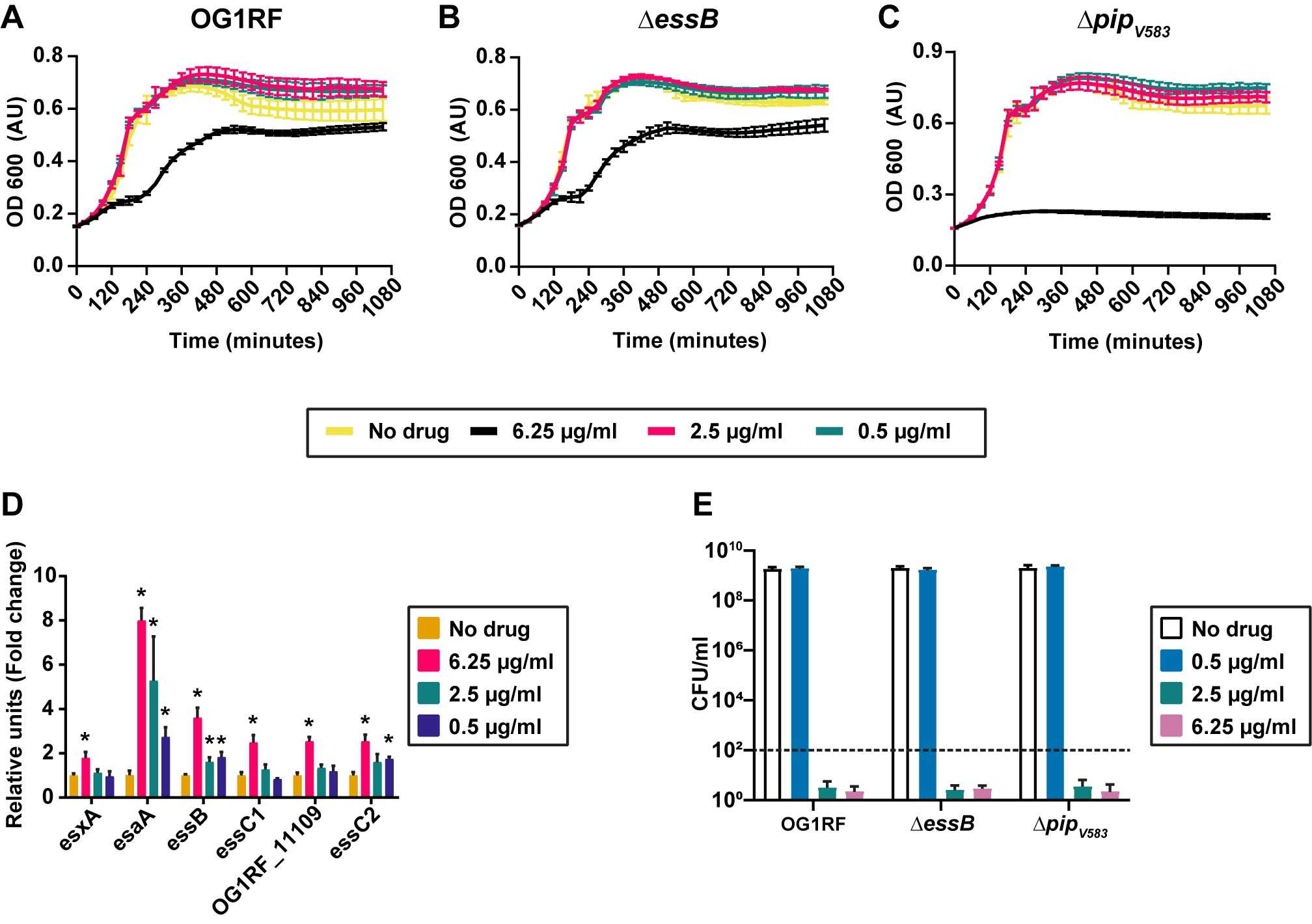

Supplement: S8 Fig — Growth of different enterococcal strains either untreated or treated with 6.25 μg/ml, 2.5 μg/ml or 0.5 μg/ml of daptomycin in (A–C) liquid media. (D) T7SS transcripts were measured from E. faecalis OG1RF cells grown in liquid media containing either no daptomycin or 6.25 μg/ml, 2.5 μg/ml, or 0.5 μg/ml of daptomycin. The data are expressed as the average of three biological replicates ± the standard deviation. P < 0.001 by unpaired Student’s t-test. (E) Viable bacterial cells recovered from growth on daptomycin supplemented agar media for 24 hours. The dashed line indicates the limit of detection. (TIF) [file pgen.1009204.s008.tif]

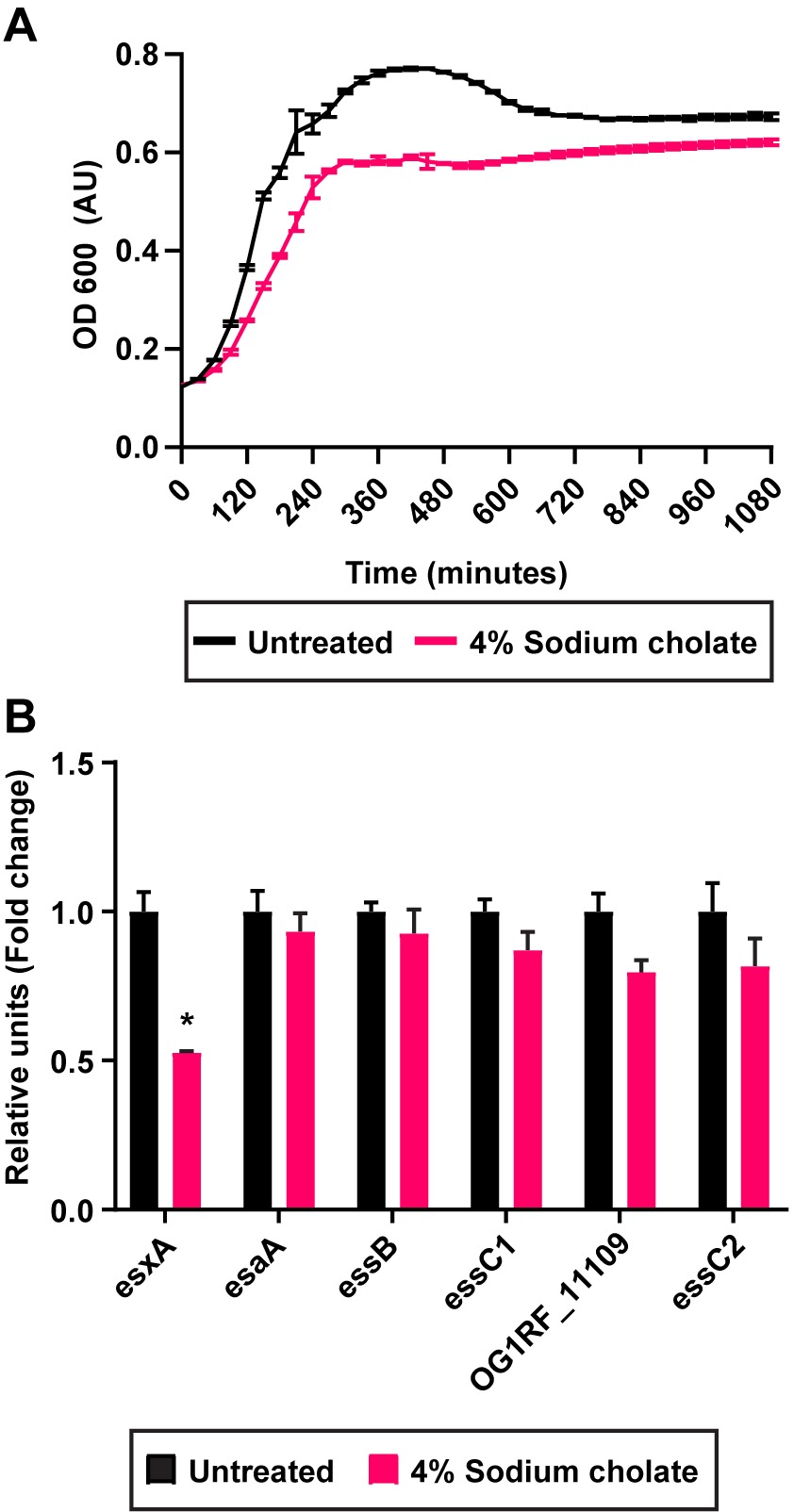

Supplement: S9 Fig — (A) Optical density of wild type E. faecalis OG1RF grown in the absence and presence of 4% sodium cholate was measured for 18 hours. (B) Transcript levels of OG1RF T7SS genes in untreated and 4% sodium cholate treated E. faecalis OG1RF after 4 hours. P < 0.001 to by unpaired Student’s t-test. Error bars indicate standard deviation. (TIF) [file pgen.1009204.s009.tif]

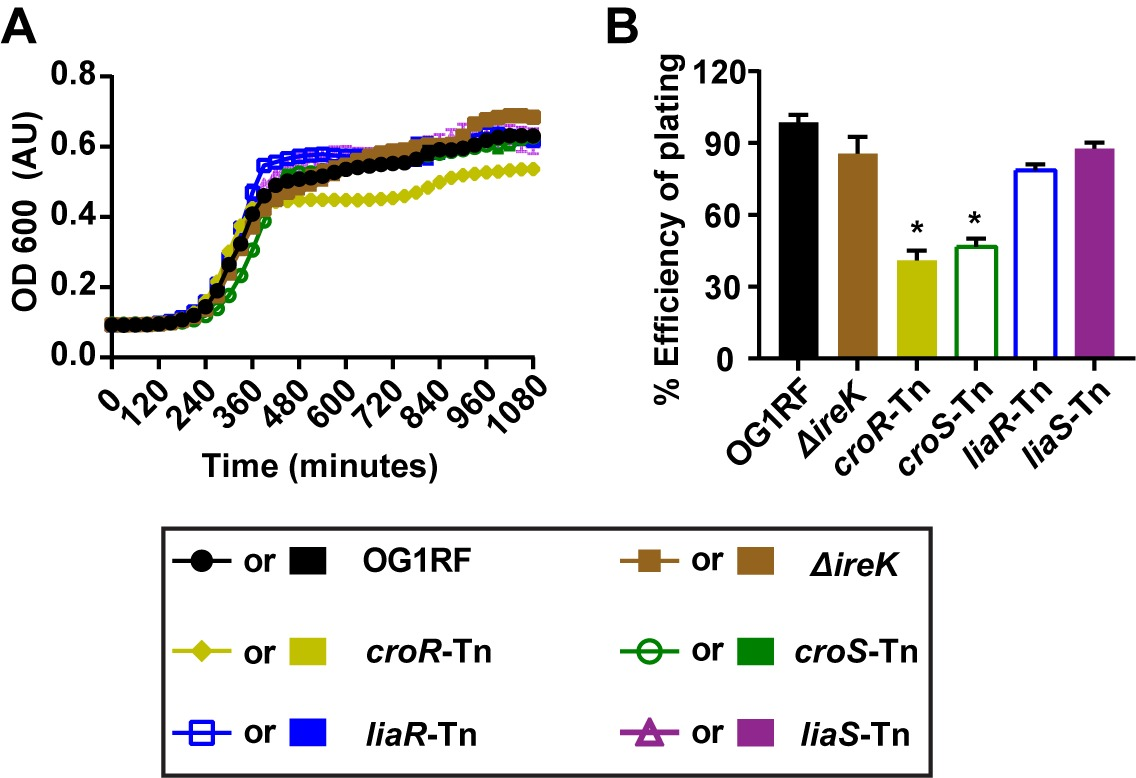

Supplement: S10 Fig — (A) Optical density of wild type E. faecalis OG1RF and isogenic mutants were monitored for 18 hours. (B) While all strains were susceptible to phage VPE25 infection, the proportion of released phage particles was diminished in the croR and croS transposon mutant background. Data represent three biological replicates. Error bars indicate standard deviation. *P < 0.001 by unpaired Student’s t-test. (TIF) [file pgen.1009204.s010.tif]

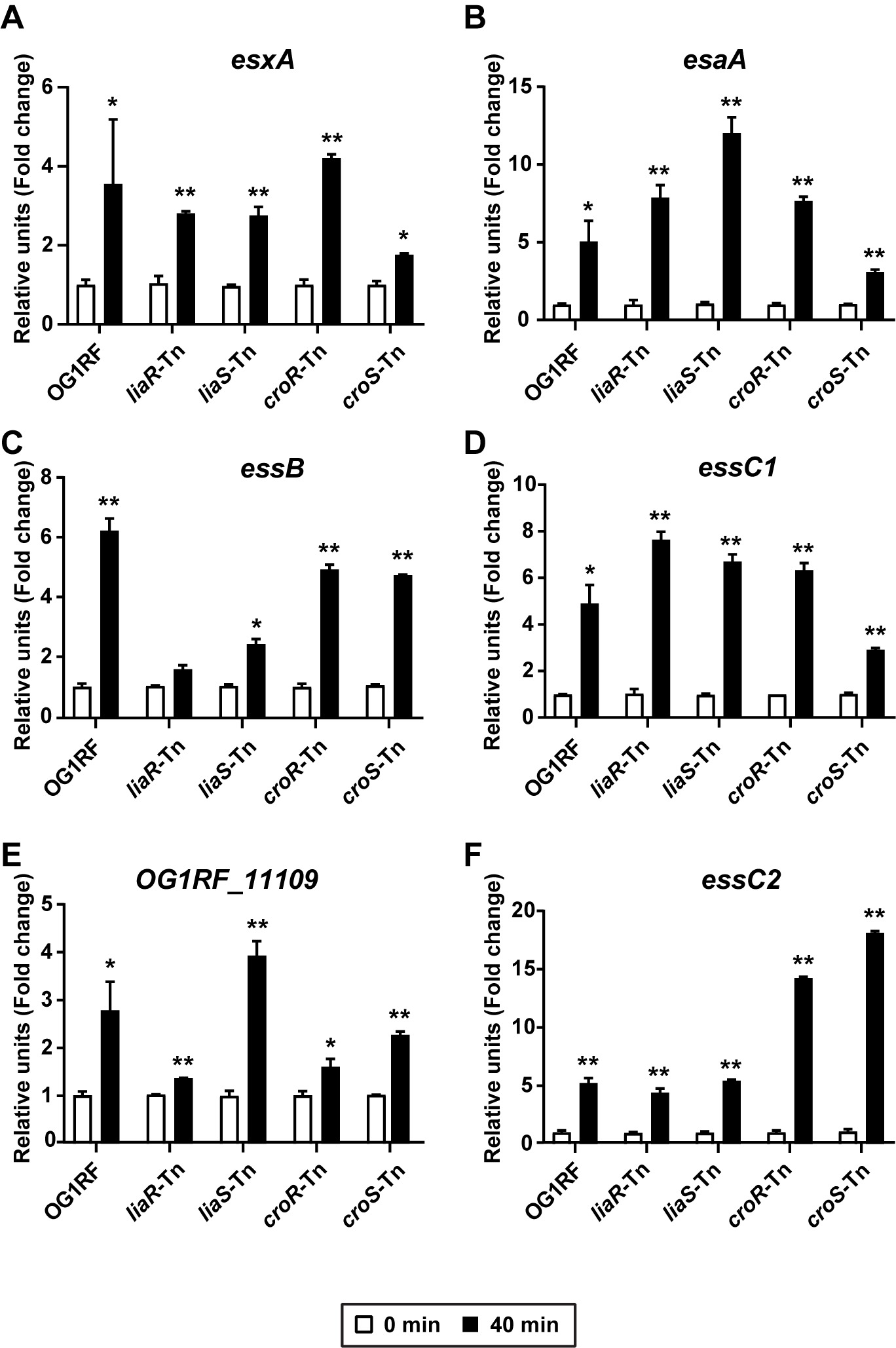

Supplement: S11 Fig — (A- F) mRNA transcript levels of T7SS genes are enhanced in the transposon mutants of liaR, liaS, croR and croS strains similar to wild type E. faecalis OG1RF during phage infection (MOI = 1) compared to untreated controls. Data represent three biological replicates. Error bars indicate standard deviation. *P < 0.01, **P < 0.0001 by unpaired Student’s t-test. (TIF) [file pgen.1009204.s011.tif]

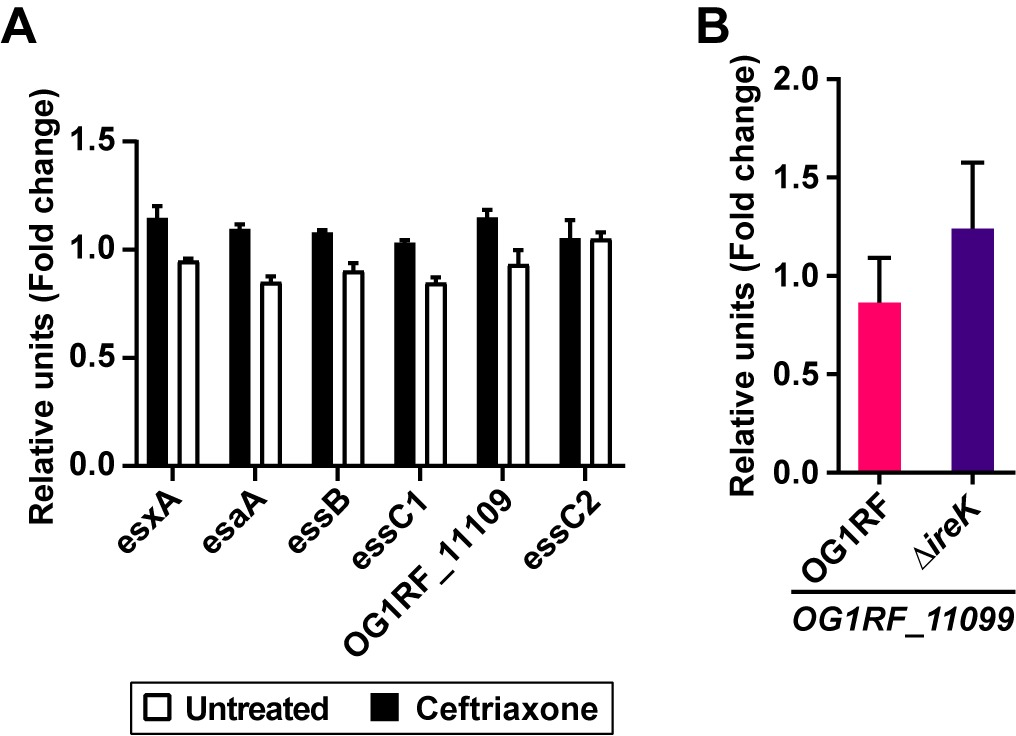

Supplement: S12 Fig — (A) Transcription of T7SS genes in E. faecalis OG1RF are not elevated 20 minutes post ceftriaxone (128μg/ml) administration relative to an untreated control. (B) OG1RF_11099 expression remains unaltered during phage predation of wild type E. faecalis OG1RF and ΔireK strains relative to uninfected controls. Data represent three biological replicates. Error bars indicate standard deviation. (TIF) [file pgen.1009204.s012.tif]
