# Supplementary material for: Phage infection and sub-lethal antibiotic exposure mediate Enterococcus faecalis type VII secretion system dependent inhibition of bystander bacteria
Source: PLoS Genet. 2021 Jan 7;17(1):e1009204. doi: 10.1371/journal.pgen.1009204 (PMC7790226; doi:10.1371/journal.pgen.1009204)
Supplement: S1 Table — (DOCX) [file pgen.1009204.s013.docx]

**Table S1. List of bacterial strains, phages, plasmids, and primers used in this study**

| **Strains, phages,**  **plasmids, and primers** | **Characteristics**  **and/or description** | **Reference**  **/Source** |
| --- | --- | --- |
| ***Enterococcus faecalis*** | | |
| OG1RF | Human oral isolate; Rf^R^, Fa^R^ | [1] |
| *∆pip*_V583_ | V583 background with a deletion of *pip*. Vm^R^, Em^R^, Gm^R^ | [2] |
| *∆ireK* | *E. faecalis* OG1RF CK119 | [3] |
| *∆essB* | *E. faecalis* OG1RF markerless deletion in OG1RF_11104 | This study |
| *croR-Tn* | *E. faecalis* OG1RF *croR* transposon mutant. Rf^R^, Fa^R^, Cm^R^ | [4] |
| *croS-Tn* | *E. faecalis* OG1RF *croS* transposon mutant. Rf^R^, Fa^R^, Cm^R^ | [4] |
| *liaR-Tn* | *E. faecalis* OG1RF *liaR* transposon mutant. Rf^R^, Fa^R^, Cm^R^ | [4] |
| *liaS-Tn* | *E. faecalis* OG1RF *liaS* transposon mutant. Rf^R^, Fa^R^, Cm^R^ | [4] |
| OG1RF (pLZ12A) | *E. faecalis* OG1RF carrying pLZ12A empty vector. Rf^R^, Fa^R^, Cm^R^ | This study |
| ∆*essB* (pLZ12A) | ∆*essB* strain carrying pLZ12A empty vector. Rf^R^, Fa^R^, Cm^R^ | This study |
| ∆*essB* (pLZ12A::*essB*) | ∆*essB* strain carrying pLZ12A complementation vector. Rf^R^, Fa^R^, Cm^R^ | This study |
| ∆*pip_V583_* (pLZ12A) | *∆pip_V583_* strain carrying pLZ12A empty vector. Vm^R^, Em^R^, Gm^R^, Cm^R^ | [5] |
| ∆*pip_V583_* (pLZ12A::*11110*) | *∆pip_V583_* strain carrying pLZ12A containing coding sequence of OG1RF_11110 from P-*bacA.* Vm^R^, Em^R^, Gm^R^, Cm^R^ | This study |
| ∆*pip_V583_* (pLZ12A::*11112*) | *∆pip_V583_* strain carrying pLZ12A containing coding sequence of OG1RF_11112 from P-*bacA.* Vm^R^, Em^R^, Gm^R^, Cm^R^ | This study |
| ∆*pip_V583_* (pLZ12A::*11122*) | *∆pip_V583_* strain carrying pLZ12A containing coding sequence of OG1RF_11122 from P-*bacA.* Vm^R^, Em^R^, Gm^R^, Cm^R^ | This study |
| ∆*pip_V583_* (pLZ12A::*12413*) | *∆pip_V583_* strain carrying pLZ12A containing coding sequence of OG1RF_12413 from P-*bacA.* Vm^R^, Em^R^, Gm^R^, Cm^R^ | This study |
| OG1RF (pCIEtm) | *E. faecalis* OG1RF carrying pCIEtm empty vector. Rf^R^, Fa^R^, Tc^R^ | This study |
| ∆*essB* (pCIEtm) | ∆*essB* strain carrying pCIEtm empty vector. Rf^R^, Fa^R^, Tc^R^ | This study |
| OG1RF_11121-Tn | *E. faecalis* OG1RF *OG1RF-11121* transposon mutant. Rf^R^, Fa^R^, Cm^R^ | [4] |
| OG1RF_11121-Tn (pCIEtm) | OG1RF_11121-Tn strain carrying pCIEtm empty vector. Rf^R^, Fa^R^, Cm^R^, Tc^R^ | This study |
| OG1RF_11121-Tn (pCIEtm::*11121*) | OG1RF_11121-Tn strain carrying pCIEtm complementation vector. Rf^R^, Fa^R^, Cm^R^, Tc^R^ | This study |
| *∆ireK* (pCIE) | *∆ireK* strain carrying pCIE empty vector. Rf^R^, Fa^R^, Cm^R^ | This study |
| *∆ireK* (pCIE::*ireK*) | *∆ireK* strain carrying pCIE complementation vector. Rf^R^, Fa^R^, Cm^R^ | This study |
| OG1RF_11099-Tn | *E. faecalis* OG1RF *OG1RF-11099* transposon mutant. Rf^R^, Fa^R^, Cm^R^ | [4] |
| OG1RF_11099-Tn (pCIEtm) | OG1RF_11099-Tn strain carrying pCIEtm empty vector. Rf^R^, Fa^R^, Cm^R^, Tc^R^ | This study |
| OG1RF_11099-Tn (pCIEtm::*11099*) | OG1RF_11099-Tn strain carrying pCIEtm complementation vector. Rf^R^, Fa^R^, Cm^R^, Tc^R^ | This study |
| **Other bacteria** | | |
| *S. aureus* | *Staphylococcus aureus* strain LAC* φ11::LL29 *tet*. Tc^R^ | [6] |
| *E. faecium* | *Enterococcus faecium* strain 1,231,410. Vm^R^, Em^R^ | [7] |
| *L. monocytogenes* | *Listeria monocytogenes* 10403S. St^R^ | [8] |
| *L. lactis* | *Lactococcus lactis* NZ9000 | Doran lab |
| *S. agalactiae* | *Streptococcus agalactiae* strain COH1. | [9] |
| *S. pyogenes* | *Streptococcus pyogenes* ATCC 12384. | ATCC |
| *S. mitis* | *Streptococcus mitis* NS5. Clinical isolate from UT Southwestern Clinical Microbiology Laboratory |  |
| *S. gordonii* | *Streptococcus gordonii*ATCC® 49818. St^R^ | Doran lab |
| *S. salivarius* | *Streptococcus salivarius* K12. Sp^R^ | Doran lab |
| *S. enterica* | *Salmonella enterica*serovar Typhimurium. AV09379 *put*::Kan; Kn^R^ | [10] |
| *V. cholerae* | *Vibrio cholerae C6706 int I4::TnFL63*; Kn^R^ | [11] |
| ***Escherichia coli*** | | |
| TG1 | *[F' traD36 proAB lacIqZ ∆M15] supE thi-1 ∆(lac-proAB) ∆(mcrBhsdSM)5(rK - mK -)* | Lucigen |
| K12 | *Escherichia coli* K12, ATCC 25404 | ATCC |
| **Phage** | | |
| VPE25 | Siphoviridae; Wastewater isolate | [2] |
| **Plasmids** | | |
| pLZ12A | *bacA* promoter cloned into shuttle vector pLZ12; pSH71 origin; Cm^R^ | [5, 12] |
| pLT06 | *E. faecalis* allelic exchange vector; Cm^R^ | [13] |
| pBD01 | *∆essB* construct cloned into pLT06 by Gibson assembly. Cm^R^ | This study |
| pLZ12A::*essB* | *essB* complementation vector. Cloned into PstI/BamHI site. Cm^R^ | This study |
| pLZ12A::*11110* | pLZ12A expressing *OG1RF_11110* from P*_bacA_*. Cloned into PstI/BamHI site. Cm^R^ | This study |
| pLZ12A::*11112* | pLZ12A expressing *OG1RF_11112* from P*_bacA_*. Cloned into PstI/BamHI site. Cm^R^ | This study |
| pLZ12A::*11122* | pLZ12A expressing *OG1RF_11122* from P*_bacA_*. Cloned into PstI/BamHI site. Cm^R^ | This study |
| pLZ12A::*12413* | pLZ12A expressing *OG1RF_11122* from P*_bacA_*. Cloned into PstI/BamHI site. Cm^R^. | This study |
| pCIE | cCF10 pheromone inducible P_Q_ expression vector. Cm^R^ | [14] |
| pCIEtm | Pheromone-inducible pCIE vector with tetracycline resistance cassette. Tet^R^ | {Willett, 2019, Exploiting biofilm phenotypes for functional characterization of hypothetical genes in Enterococcus faecalis}[15] |
| pCIEtm::*11121-11122* | pCIEtm expressing OG1RF_11121-11122 from cCF10 responsive promoter P_Q_ (11122 is also under control of native promoter). Cloned with BamHI/XbaI into BamHI/NheI sites. Tet^R^. | This study |
| pCIEtm::*11099* | pCIEtm expressing OG1RF_11099 from cCF10 responsive promoter P_Q_. Cloned into BamHI/PvuI site. Tet^R^ | This study |
| pGEM-T-Easy | Cloning vector, Amp^R^ | Promega |
| pGEM-T-Easy::*ireK* | pGem-T-Easy with *ireK* inserted at the T overhang. Amp^R^. | This study |
| pCIE::*ireK* | pCIEtm expressing *ireK* from cCF10 responsive promoter P_Q_. Cloned into BamHI/SphI sites. Cm^R^. | This study |
| **Primers** | | |
| *essB*-F | NNNNNNCTGCAGATGAGCGATTAAAGGATATTTCA; Forward primer to generate pLZ12A::*essB*; PstI site | This study |
| *essB*-R | NNNNNNGGATCCTTACTATTTTCGTTGTCATCC; Reverse primer to generate pLZ12A::*essB*; BamHI site | This study |
| *OG1RF_11110*-F | NNNNNNCTGCAGATGGACTTCCAAGGTGGTAAAATTAT; Forward primer to generate pLZ12A::*11110*; PstI site | This study |
| *OG1RF_11110*-R | NNNNNNGGATCCTTATTCTCCGTACCATTCCTCTTTA; Reverse primer to generate pLZ12A::*11110*; BamHI site | This study |
| *OG1RF_11112*-F | NNNNNNCTGCAGATGAATAAAATCTTAAATAAAATATCTTTTG; Forward primer to generate pLZ12A::*11112*; PstI site | This study |
| *OG1RF_11112*-R | NNNNNNGGATCCCTAACTATCTTCACCATACCATTCTTG; Reverse primer to generate pLZ12A::*11112*; BamHI site | This study |
| *OG1RF_11122*-F | NNNNNNCTGCAGATGGTTTTCATGATAAAAAATTATGTACC; Forward primer to generate pLZ12A::*11122*; PstI site | This study |
| *OG1RF_11122*-R | NNNNNNGGATCCTTATTTTTTGGTTCTCTTGTTCTTC; Reverse primer to generate pLZ12A::*11122*; BamHI site | This study |
| *11121*-bam-fwd | ATAGGATCCACGTATGTCTAATGAGGAGG; forward primer to amplify OG1RF_11121-11122; BamHI site | This study |
| *11122*-xba-rev | ATATCTAGATTATTTTTTGGTTCTCTTGTTC; reverse primer to amplify OG1RF_11122; XbaI site | This study |
| *ireK*-BamHI-F | GGATCCACCGTGTTAGTGATACA; forward primer to amplify *ireK*; BamHI site | This study |
| *ireK*-SphI-R | GCATGCTTAATTACTCGTACTACT; reverse primer to amplify *ireK*, SphI site | This study |
| *OG1RF_11099*-F | NNNNNNGGATCCATGGTTCAAAATATACCAATTTATATTCAAATTCACG; Forward primer to generate pCIEtm::11099; BamHI site | This study |
| *OG1RF_11099-R* | NNNNNNCGATCGCTACTTCTCTAAATAAAACTCAAATCGACTTCCTGC; Reverse primer to generate pCIEtm::11099; PvuI site | This study |
| *12413*-pst-fwd | ATACTGCAGTAACTATTTTAGGTTCCAGTCC; Forward primer to amplify OG1RF_12413; PstI site | This study |
| *12413*-bam-rev | TATGGATCCAAAGTATCTGGTATTGTGTTTGC; Reverse primer to amplify OG1RF_12413; BamHI site | This study |
| RT-*esxA*-F | AAGGGCAAGCATTTCAAGCG; qPCR forward primer for OG1RF_11100 | [16] |
| RT-*esxA*-R | TCTTGACGGTCACGTTCTGC; qPCR reverse primer for OG1RF_11100 | [16] |
| RT-*esaA*-F | CCAATGGCTTGGCAACTGAC; qPCR forward primer for OG1RF_11101 | [16] |
| RT-*esaA*-R | GCGAACGAACGTGCATTTTG; qPCR reverse primer for OG1RF_11101 | [16] |
| RT-*essB*-F | GGGAATGGCACCCTGAAAGA; qPCR forward primer for OG1RF_11104 | [16] |
| RT-*essB* -R | CTTCGCGCTTGGCTTTTTGA; qPCR reverse primer for OG1RF_11104 | [16] |
| RT-*essC1*-F | TTGGAAAGGTGGCGGAATAG; qPCR forward primer for OG1RF_11105 | [16] |
| RT-*essC1*-R | TCTGCTTTGATACTGGCTAAGG; qPCR reverse primer for OG1RF_11105 | [16] |
| RT-*11109*-F | GCTTTGGAGAACGCTGAACG; qPCR forward primer for OG1RF_11109 | [16] |
| RT-*11109*-R | TTTTGACAGTCTTGCGCTCG; qPCR reverse primer for OG1RF_11109 | [16] |
| RT-*essC2*-F | CTCAACCGGATCGTGCTTATT; qPCR forward primer for OG1RF_11115 | [16] |
| RT-*essC2*-R | CCTTGGTAGCGAATGGATCATAG; qPCR reverse primer for OG1RF_11115 | [16] |
| RT-*clpX*-F | ATTGGACCAACAGGTTCAGG; qPCR forward primer for *clpX* | This study |
| RT-*clpX*-R | TTTCCGCACGTTCAACATTA; qPCR reverse primer for *clpX* | This study |
| RT-11099-F | GGAACGTATGTAGCACGTAAGA; qPCR forward primer for OG1RF_11099 | This study |
| RT-11099-R | TAAGACACCGTCCGACTAGAA; qPCR reverse primer for OG1RF_11099 | This study |
| RT-16S-F | CGCTTCTTTCCTCCCGAGT; qPCR forward primer 16S rRNA gene | [16] |
| RT-16S-F | GCCATGCGGCATAAACTG; qPCR reverse primer 16S rRNA gene | [16] |

Cm^R^ - chloramphenicol resistant; Rf^R^ - rifampicin resistance; Fa^R^ - fusidic acid resistance; Vm^R^ - vancomycin resistance; Em^R^ - erythromycin resistance; Gm^R^ - Gentamicin resistance; Tc^R^ - tetracycline resistance; St^R^ = streptomycin resistance; Kn^R^ = Kanamycin resistance; Sp^R^ = spectinomycin resistance. Restriction enzyme sites are underlined.

1. Bourgogne A, Garsin DA, Qin X, Singh KV, Sillanpaa J, Yerrapragada S, et al. Large scale variation in *Enterococcus faecalis* illustrated by the genome analysis of strain OG1RF. Genome Biol. 2008;9(7):R110. Epub 2008/07/10. doi: 10.1186/gb-2008-9-7-r110. PubMed PMID: 18611278; PubMed Central PMCID: PMCPMC2530867.

2. Duerkop BA, Huo W, Bhardwaj P, Palmer KL, Hooper LV. Molecular basis for lytic bacteriophage resistance in enterococci. MBio. 2016;7(4). Epub 2016/09/01. doi: 10.1128/mBio.01304-16. PubMed PMID: 27578757; PubMed Central PMCID: PMCPMC4999554.

3. Kristich CJ, Wells CL, Dunny GM. A eukaryotic-type Ser/Thr kinase in *Enterococcus faecalis* mediates antimicrobial resistance and intestinal persistence. Proc Natl Acad Sci U S A. 2007;104(9):3508-13. Epub 2007/03/16. doi: 10.1073/pnas.0608742104. PubMed PMID: 17360674; PubMed Central PMCID: PMCPMC1805595.

4. Dale JL, Beckman KB, Willett JLE, Nilson JL, Palani NP, Baller JA, et al. Comprehensive functional analysis of the *Enterococcus faecalis* core genome using an ordered, sequence-defined collection of insertional mutations in strain OG1RF. mSystems. 2018;3(5). Epub 2018/09/19. doi: 10.1128/mSystems.00062-18. PubMed PMID: 30225373; PubMed Central PMCID: PMCPMC6134198.

5. Chatterjee A, Johnson CN, Luong P, Hullahalli K, McBride SW, Schubert AM, et al. Bacteriophage resistance alters antibiotic-mediated intestinal expansion of enterococci. Infect Immun. 2019;87(6):e00085-19. Epub 2019/04/03. doi: 10.1128/IAI.00085-19. PubMed PMID: 30936157; PubMed Central PMCID: PMCPMC6529655.

6. Kwiecinski JM, Crosby HA, Valotteau C, Hippensteel JA, Nayak MK, Chauhan AK, et al. *Staphylococcus aureus* adhesion in endovascular infections is controlled by the ArlRS-MgrA signaling cascade. PLoS Pathog. 2019;15(5):e1007800. Epub 2019/05/23. doi: 10.1371/journal.ppat.1007800. PubMed PMID: 31116795; PubMed Central PMCID: PMCPMC6548404.

7. Palmer KL, Carniol K, Manson JM, Heiman D, Shea T, Young S, et al. High-quality draft genome sequences of 28 *Enterococcus* sp. isolates. J Bacteriol. 2010;192(9):2469-70. Epub 2010/03/09. doi: 10.1128/JB.00153-10. PubMed PMID: 20207762; PubMed Central PMCID: PMCPMC2863476.

8. Rayamajhi M, Humann J, Penheiter K, Andreasen K, Lenz LL. Induction of IFN-alphabeta enables *Listeria monocytogenes* to suppress macrophage activation by IFN-gamma. J Exp Med. 2010;207(2):327-37. Epub 2010/02/04. doi: 10.1084/jem.20091746. PubMed PMID: 20123961; PubMed Central PMCID: PMCPMC2822610.

9. Kuypers JM, Heggen LM, Rubens CE. Molecular analysis of a region of the group B streptococcus chromosome involved in type III capsule expression. Infect Immun. 1989;57(10):3058-65. Epub 1989/10/01. PubMed PMID: 2550369; PubMed Central PMCID: PMCPMC260770.

10. Fitzsimmons LF, Liu L, Kant S, Kim JS, Till JK, Jones-Carson J, et al. SpoT induces intracellular *Salmonella* virulence programs in the phagosome. mBio. 2020;11(1). Epub 2020/02/27. doi: 10.1128/mBio.03397-19. PubMed PMID: 32098823; PubMed Central PMCID: PMCPMC7042702.

11. Cameron DE, Urbach JM, Mekalanos JJ. A defined transposon mutant library and its use in identifying motility genes in *Vibrio cholerae*. Proc Natl Acad Sci U S A. 2008;105(25):8736-41. Epub 2008/06/25. doi: 10.1073/pnas.0803281105. PubMed PMID: 18574146; PubMed Central PMCID: PMCPMC2438431.

12. Perez-Casal J, Caparon MG, Scott JR. Mry, a trans-acting positive regulator of the M protein gene of *Streptococcus pyogenes* with similarity to the receptor proteins of two-component regulatory systems. J Bacteriol. 1991;173(8):2617-24. Epub 1991/04/01. PubMed PMID: 1849511; PubMed Central PMCID: PMCPMC207828.

13. Thurlow LR, Thomas VC, Hancock LE. Capsular polysaccharide production in *Enterococcus faecalis* and contribution of CpsF to capsule serospecificity. J Bacteriol. 2009;191(20):6203-10. Epub 2009/08/18. doi: 10.1128/JB.00592-09. PubMed PMID: 19684130; PubMed Central PMCID: PMCPMC2753019.

14. Weaver KE, Chen Y, Miiller EM, Johnson JN, Dangler AA, Manias DA, et al. Examination of *Enterococcus faecalis* toxin-antitoxin system toxin Fst function utilizing a pheromone-inducible expression vector with tight repression and broad dynamic range. J Bacteriol. 2017;199(12). Epub 2017/03/30. doi: 10.1128/JB.00065-17. PubMed PMID: 28348028; PubMed Central PMCID: PMCPMC5446624.

15. Willett JL, Ji M, Dunny GM. Exploiting biofilm phenotypes for functional characterization of hypothetical genes in *Enterococcus faecalis*. npj Biofilms and Microbiomes volume2019.

16. Chatterjee A, Willett JLE, Nguyen UT, Monogue B, Palmer KL, Dunny GM, et al. Parallel genomics uncover novel enterococcal-bacteriophage interactions. mBio. 2020;11(2). Epub 2020/03/05. doi: 10.1128/mBio.03120-19. PubMed PMID: 32127456; PubMed Central PMCID: PMCPMC7064774.
